# Combined Transcriptomic Analysis and RNA Interference Reveal the Effects of Methoxyfenozide on Ecdysone Signaling Pathway of *Spodoptera exigua*

**DOI:** 10.3390/ijms22169080

**Published:** 2021-08-23

**Authors:** Zhixian Zhang, Yajie Ma, Xiaoyan Ma, Hongyan Hu, Dan Wang, Xianpeng Song, Xiangliang Ren, Yan Ma

**Affiliations:** 1 Zhengzhou Research Base, State Key Laboratory of Cotton Biology, Zhengzhou University, Zhengzhou 450001, China; zhangzhixianmhs@163.com (Z.Z.); harmani@126.com (X.M.); 2 State Key Laboratory of Cotton Biology, Institute of Cotton Research, Chinese Academy of Agricultural Sciences, Anyang 455000, China; yun1268486@126.com (Y.M.); huhongyan1986@163.com (H.H.); nywangdan@sina.com (D.W.); sxp15294@126.com (X.S.)

**Keywords:** *Spodoptera exigua*, methoxyfenozide, toxicity, ecdysone receptor A, mortality

## Abstract

*Spodoptera exigua* is a worldwide pest afflicting edible vegetables and has developed varying levels of resistance to insecticides. Methoxyfenozide (MET), an ecdysteroid agonist, is effective against lepidopteran pests such as *S. exigua*. However, the mechanism of MET to *S. exigua* remains unclear. In this study, we analyzed the expression patterns of genes related to the ecdysone signaling pathway in transcriptome data treated with sublethal doses of MET and analyzed how expression levels of key genes affect the toxicity of MET on *S. exigua*. Our results demonstrated that 2639 genes were up-regulated and 2512 genes were down-regulated in *S. exigua* treated with LC_30_ of MET. Of these, 15 genes were involved in the ecdysone signaling pathway. qPCR results demonstrated that *ecdysone receptor A* (*EcRA*) expression levels significantly increased in *S. exigua* when treated with different doses of MET, and that the RNAi-mediated silencing of *EcRA* significantly increased mortality to 55.43% at 72 h when L3 *S. exigua* larvae were exposed to MET at the LC_30_ dose. Additionally, knocking down *EcRA* suppressed the most genes expressed in the ecdysone signaling pathway. The combination of MET and ds*EcRA* affected the expression of *E74* and enhanced the expression of *TREA*. These results demonstrate that the adverse effects of sublethal MET disturb the ecdysone signaling pathway in *S. exigua*, and *EcRA* is closely related to MET toxic effect. This study increases our collective understanding of the mechanisms of MET in insect pests.

## Highlights

Five genes were up-regulated and 10 genes were down-regulated in *Spodoptera exigua* treated with LC_30_ of Methoxyfenozide, all of which were involved in the ecdysone signaling pathway.

The expression of *EcRA* was up-regulated as treatments of Methoxyfenozide on *S. exigua* increased.

Knockdown of *EcRA* expression by RNAi dramatically increases the sensitivity of *S. exigua* to Methoxyfenozide.

Knockdown of *EcRA* expression affects the expression of the ecdysone signaling pathway genes in Methoxyfenozide-treated *S. exigua*. 

## 1. Introduction

*Spodoptera exigua* (Lepidoptera, Noctuidae) is a polyphagous pest. It attacks important cultivated and industrial crops, including cotton and sugar beets, resulting in significant losses [1]. Therefore, controlling *S. exigua* is important for agricultural production, and insecticides can be used for this purpose [2,3,4]. Traditional chemical insecticides are the primary method of controlling *S. exigua* [2,3,4]. However, the doses of traditional chemical controls increase with time and can damage the crops [5]. Integrated Pest Management (IPM) is a widely used method of controlling insect pests, while Insect Growth Regulators (IGRs) play a pivotal role in IPM [6]. Compared with traditional chemical controls, IGRs have specific advantages: they produce less residue, are easily degradable, and can induce insect molting which results in death [7].

IGRs contain Juvenile Hormone agonists (JH), ecdysteroid agonists (20E), and Chitin Biosynthesis Inhibitors (CSI). 20E is a molting hormone and combines with the ecdysone receptor (EcR) and ultraspiracle (USP), turning into a compound [8]. This compound triggers the expression of the 20E response gene. The research literature shows that ecdysone signaling pathway involves in *EcR*, *USP*, and several other ecdysone-responsive genes, including the *ecdysone-induced protein 74EF* (*E74*), nuclear *hormone receptor HR3* (*HR3*), *hormone receptor 4* (*HR4*), *hormone receptor FTZ-F1* (*FTZ-F1*), *hormone receptor HR38* (*HR38*), and chitin biosynthesis pathway genes [9,10,11,12]. The chitin biosynthesis pathway contains *Trehalose 6-phosphate* (*TPS*), *Trehalase* (*TRE*), *Hexokinase* (*HK*), *Glucose-6-phosphate isomerase* (*G6PI*), *Glutamine fructose-6-phosphate aminotransferase* (*GFAT*), *Phosphoacetyl Glucosamine Mutase* (*PGM*), *N-acetyltransferase UDP-N-acetylglucosamine Pyrophosphorylase* (*UAP*), and *Chitin Synthase* (*CHS*), all of which have been found in several insect species [13,14,15,16].

In Lepidoptera, the prothoracicotropic hormone (PTTH)-stimulated ecdysteroidogenesis and larval growth are involved in two ways, mitogen-activated protein kinase (MAPK) and phosphoinositide 3-kinase/protein kinase B/target of rapamycin (PI3K/Akt/TOR) pathways [14,17,18]. Ecdysone is subsequently activated to 20-OH-ecdysone in peripheral tissues [17]. 20E binds to a heterodimer of the EcR and USP, and the 20E-EcR/USP complex controls the transcription of target genes [8]. EcR is an ecdysone receptor and has been isolated from many insect species. *Helicoverpa armigera* that were fed *Escherichia coli* expressing amiRNA-Ha*EcR* exhibited developmental defects and high rates of mortality [12]. The suppression of Se*EcR* influenced developmental duration, pupae formation, adult eclosion, and the chitin content of *S. exigua* [19]. To elucidate the role of *EcR* in physiological processes, the relationship between *EcR* and insecticides has been reported. In *Conopomorpha sinensis*, *EcR* expression was changed by chlorbenzuron [20]. Treating *S. exigua* with tebufenozide or RH-5849 demonstrated that ecdysone agonists binding with *EcR* [21]. After treatment with Methoxyfenozide (MET), *EcR* expression was enhanced, and subsequently impaired fecundity [22]. MET is an ecdysteroid agonist and can combine with EcR compounds, leading to lethal molting [23]. Therefore, some studies assessed the toxicity of *S. exigua* treated with MET [24,25]. However, few studies have assessed how MET affects the ecdysone signaling pathway genes and the mechanism for MET toxicity in *S. exigua*.

In this study, the transcriptome of *S. exigua* fed with MET were sequenced using an Illumina TruseqTM RNA Sample Prep Kit. We determined that the ecdysone signaling pathway genes associated with *S. exigua* fed with MET. Using quantitative real-time PCR (qPCR), identified the gene expression change and used RNAi to assess the role that *EcRA* involve in increasing *S. exigua* sensitivity to MET. We also assessed the expression patterns of ecdysone signaling pathway gene changes in MET and RNAi treated larvae. These results reveal different doses of MET affects gene expression on ecdysone signaling pathway, exhibiting that knocking down *EcRA* impacts mortality of MET and the expression of ecdysone signaling pathway genes of *S. exigu*. This study attempts to determine the interaction of *EcRA* and MET on *S. exigua*, and further explore the action mechanisms of MET.

## 2. Results

### 2.1. Transcriptome Data Analysis

**Figure 1:**
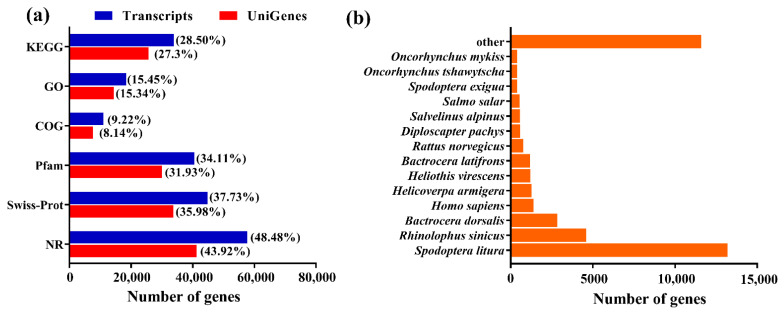
Gene annotation and homology search. (**a**) The number of genes using six databases. NR, Swiss-Prot, Pfam, COG, GO, and KEGG. (**b**) Homology search of Illumina sequences matching with the NR database. 18,935 NR-annotated transcripts sequences were analyzed for species distribution.

In order to identify the genes involved in the ecdysone signaling pathway, transcriptome sequencing analysis was used to sequence the transcriptomes of *S. exigua* larvae in MET and control groups. Quality control was performed on the original sequencing data to obtain high-quality data and conduct statistical analyses (Table 1). The transcriptome sequences were connected, filtered, and optimized using TransRate and Trinity. The two group transcriptome sequencing results were completed, after which the bioinformatics analysis was performed. The relativity of the sample data indicated that the results of the transcriptome sequencing were viable. The UniGenes and Transcripts number were successfully annotated in *S. exigua* (Figure 1a). *Spodoptera litura* had the highest homology compared with *S. exigua* (Figure 1b). 

The total number of transcripts and UniGenes were 118,834 and 93,874. After filtering, the average error rate located with 0.02~0.03%. The results show that >98% of each sample had a quality score of Q20, and >95% of each sample had a quality score of Q30. The GC content of the *S. exigua* were more than 49% (control group) and 49% (MET).

**Figure 2:**
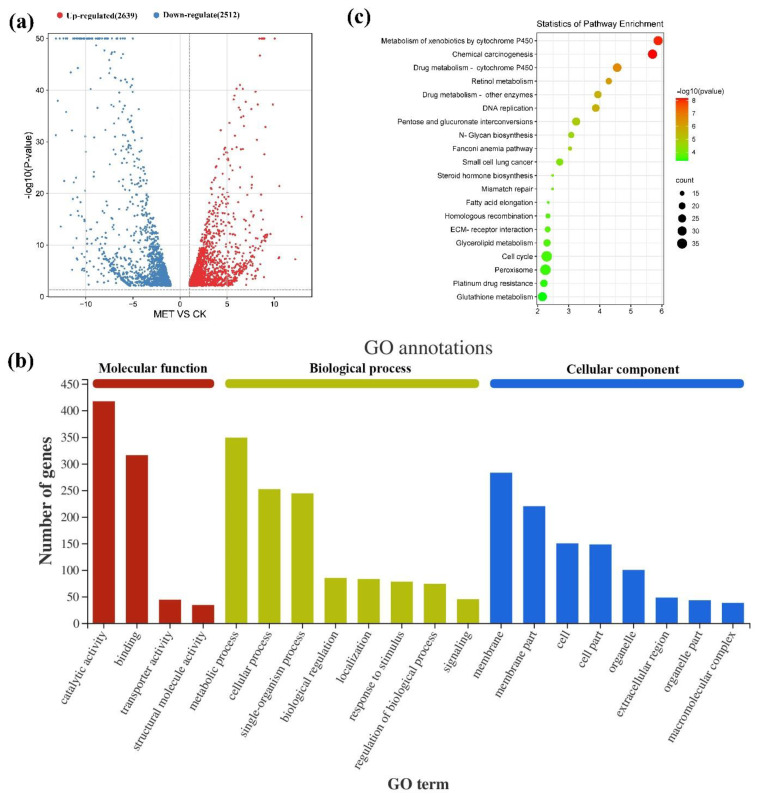
Function annotation and enrichment of DEGs. (**a**) Volcano plot of differentially expressed genes of *S. exigua* fed with MET (red spots represent significantly up-regulated genes; green spots represent significantly down-regulated genes). (**b**) GO function annotation analysis of *S. exigua* fed with MET (metabolic process under biological process had the primary number of UniGenes. The catalytic activity and binding under the molecular function were the most dominant. The membrane under the cellular component were the biggest group). (**c**) The most enriched KEGG pathways of *S. exigua* after exposure to a sublethal concentration of MET.

The functional distribution of differentially expressed genes (DEGs) in *S. exigua* was determined by comparing its transcriptomes in response to MET. Compared with the control group, 2639 genes were up-regulated and 2512 genes were down-regulated (Figure 2a). According to the GO terms, 3798 UniGenes were divided into three categories (biological processes, molecular functions, and cellular components) containing 20 variety classes. Catalytic activity (167), binding (104), and metabolic processes (88) contained the most UniGenes in the three categories (Figure 2b). By comparing the up-regulated and down-regulated GO enrichment, it was apparent that nearly all of them in *S. exigua* were more up-regulated than down-regulated after exposure to the sublethal dose of MET (Figure S1). KEGG enrichment showed that 20 KEGG pathways were assigned to signification enrichment (Figure 2c). There were 15 genes involved in the ecdysone signaling pathway (Table 2). In qPCR, *EcRA*, *USP*, and *HR3* were up-regulated and *E74*, *HR4*, *FTZ-F1*, and *HR38* were down-regulated. The qPCR results were compared with the RNA-seq analysis, indicating that the RNA-seq results were reliable (Figure S2).

### 2.2. Sublethal Doses of MET Induction on Ecdysone Signaling Pathway

**Figure 3:**
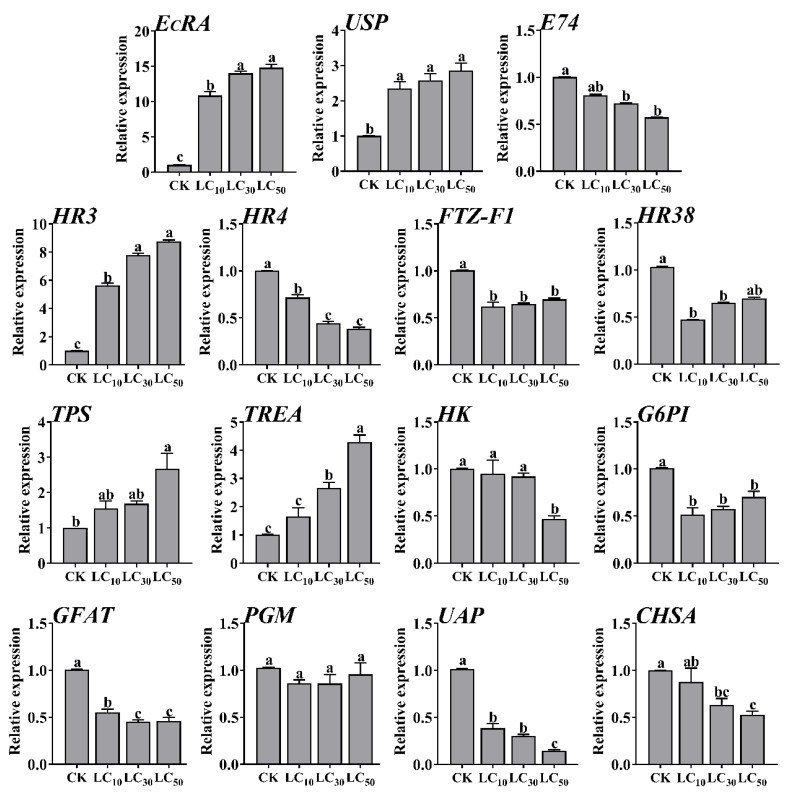
Relative expression levels of ecdysone signaling pathway genes in *S. exigua* under MET treatment. Relative expression levels of ecdysone signaling pathway in L3 larvae were examined 72 h after exposure to the control treatment DEPC water (CK) or MET at three different dosages (LC_10_, LC_30_, and LC_50_). The bars represent the average (±SE) of biological repeats. Different letters above the bars indicate significant differences (*p* < 0.05), based on one-way ANOVA and at least significant difference test.

Different doses (LC_10_, LC_30_, and LC_50_) of MET were used to examine the dose-dependent effects in ecdysone signaling pathway gene expression in *S. exigua. EcRA*, *USP*, and *HR3* were positively up-regulated and *E74*, *HR4*, *FTZ-F1*, and *HR38* were down-regulated after the larvae were treated with different doses of MET. Notably, the up-regulation of *EcRA* increased to 10.85-fold under LC_10_ treatment, 14.02-fold under LC_30_ treatment, and 14.81-fold under LC_50_ treatment (Figure 3)*. TPS* and *TREA* were up-regulated and *HK*, *G6PI*, *GFAT*, *UAP*, and *CHSA* were down-regulated after *S. exigua* were treated with different doses of MET. The up-regulation of *TREA* increased 1.65-fold under LC_10_ treatment, 2.66-fold under LC_30_ treatment, and 4.28-fold under LC_50_ treatment (Figure 3).

### 2.3. Down-Regulation of EcRA Increase the Mortality of MET to S. exigua

**Figure 4:**
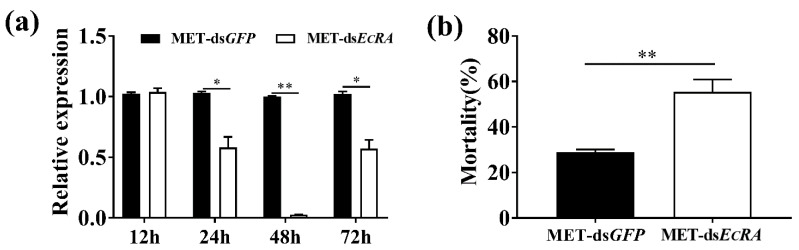
Knockdown of *EcRA* increases the sensitivity of *S. exigua* to MET. (**a**) Relative expression of *EcRA*; (**b**) mortality (%) of the ds*EcRA*-treated *S. exigua* at 72 h after MET treatment. The columns represent averages, with vertical lines indicating SE. Significant differences were calculated by Student’s *t*-test (* *p* < 0.05; ** *p* < 0.01).

In order to explore the role of *EcRA* in toxicity of MET, we knocked down *EcRA* and interfering efficiency in epidermis was examined. Our results showed that the relative transcription levels of *EcRA* did not significantly change after treatment with MET at 12 h. However, the transcripts of *EcRA* were attenuated from 24 h to 72 h after RNAi (Figure 4a). The lowest expression level of *EcRA* was found at 48 h, with an 0.03-fold to the ds*GFP*. In addition, a nonsignificant benefit in mortality was observed when compared with ds*GFP* after the ds*EcRA* was injected at 24 h, 48 h, and 72 h (not shown). Knock down of *EcRA* in L3 larvae resulted in increased mortality. Approximately 55.43% of MET-ds*EcRA* treated larvae at the LC_30_ dose of MET (Figure 4b).

### 2.4. The Expression of Ecdysone Signaling Pathway Genes Are Regulated by MET-dsEcRA

**Figure 5:**
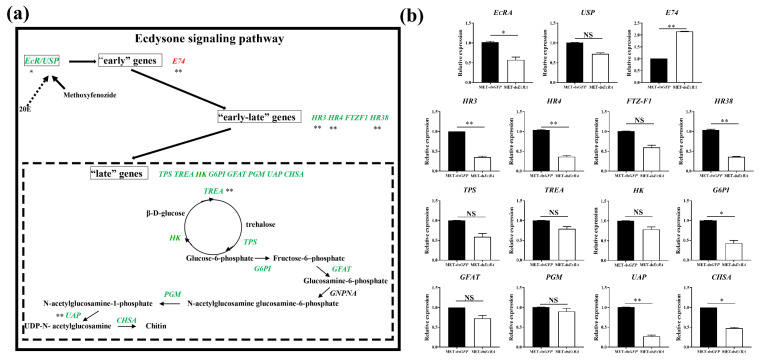
Ecdysone signaling pathway in insects. (**a**) 20E can bind to combine EcRA and USP, but MET can advance combination with EcRA and result in lethal molting. The compound could activate downstream genes. Downstream genes contain three category genes. The early genes contain *E74*, while early–late genes contain *HR3*, *HR4*, *FTZ-F1*, and *HR38*. Late genes contain chitin synthesis genes. Β-d-glucose is converted to Glucose-6-phosphate, Fructose-6-phosphate, Glucosamine-6-phosphate, N-acetylglucosamine glucosamine-6-phosphate, N-acetylglucosamine-1-phosphate, UDP-N- acetylglucosamine, and then to chitin. The activity of the enzymes encoded by *TPS*, *TRE*, *HK*, *G6PI*, *GFAT*, *GNPNA*, *PGM*, *UAP*, and *CHSA*. Significant differences were calculated by Student’s *t*-test (* *p* < 0.05; ** *p* < 0.01). (**b**) Relative expression of ecdysone signaling pathway genes after they were injected with ds*EcRA* in *S. exigua* under MET treatment. Columns represent averages, with vertical lines indicating SE. Significant differences were calculated by Student’s *t*-test (NS, *p* > 0.05; * *p* < 0.05; ** *p* < 0.01).

We collected the survival larvae treated with MET-ds*EcRA* at 72 h, the relative transcription levels of ecdysone signaling pathway genes were checked by qPCR. It was found that knock down of *EcRA* could influence other genes in the ecdysone signaling pathway of *S. exigua* (Figure 5a). The expression levels of *HR3, HR4*, and *HR38* could be down-regulated by 0.34-fold, 0.36-fold, and 0.37-fold, respectively, after the larvae treated with MET-ds*EcRA*. The expression levels of *E74* were up-regulated 2.15-fold, while there was no change in gene expression of *USP* and *FTZ-F1*. Moreover, down-regulation of *EcRA* affected expression of chitin biosynthesis pathway genes. The expression levels of *G6PI*, *UAP*, and *CHSA* were down-regulated by 0.42-fold, 0.26-fold, and 0.48-fold, respectively (Figure 5b).

## 3. Discussion

*S. exigua* (Lepidoptera, Noctuidae) is a polyphagous pest. It attacks high value cultivated and industrial crops, including cotton and sugar beets [1]. Therefore, some studies assessed the toxicity of *S. exigua* treated with MET [24,25]. However, there are few studies assessing the relationship between MET toxicity and the role of genes in *S. exigua*. In this study, we analyzed *S. exigua* after it was treated with MET to assess the role of genes involved in the toxicity of MET. The result of the transcriptome sequencing, DEGs, qPCR, RNAi, and bioassay analysis exhibited that sublethal doses of MET could adversely affect the ecdysone signaling pathway, and knock down of *EcRA* increased the mortality of MET to *S. exigua*. 

Transcriptome sequencing is used to identify the relationship between insecticides, pests, and key genes involved in pest control. Analysis of the transcriptome sequencing of Lufenuron against *Spodoptera frugiperda* provided several genes for defensive mechanisms [26]. We found 15 genes in the ecdysone signaling pathway of *S. exigua* using transcriptome sequencing. This lays a foundation for studying the relationship between insecticides and ecdysone signaling pathway genes. *EcR* and *SfUSP-2* were significantly induced by the hormone agonist MET [27]. The expression of *PxTre* increased in flubendiamide [28]. The expression of *HK*, *G6PI2*, *GFAT*, *PAGM1*, and *UAP* genes decreased significantly after the injection of validamycin [29], while mRNA levels of *ChsA* dramatically decreased in response to chlorantraniliprole [30]. MET affects development and changes the expression of the ecdysone signaling pathway of *S. exigua* [25]. Previous studies also have demonstrated that MET affected the expression of *H. armigera* 20E-related genes [31]. Our results confirmed the inductive effect of MET on the ecdysone signaling pathway. This conclusion is similar to the effect that halofenozide has on 20E-response genes in *Leptinotarsa decemlineata*, as halofenozide can activate detoxification, and the ability of agonists to regulate the expression of the 20E-response genes [32]. It has been shown that sublethal doses of MET lowered the intrinsic 20E titer [31]; therefore, MET affects larval development due to the change it induces in the intrinsic 20E titer. In our research, *EcRA* was significantly regulated in three different sublethal doses. Interestingly, *Plutella xylostella* was treated with LC_25_ and LC_50_ of fufenozide for 48 h, and *PxEcR-B* expression was downregulated [33]. *EcR* expression trends in the two species of Lepidoptera differed following treatment with the same kind of insecticides. Hence, we identified the *EcRA* gene as the primary research objective after treating *S. exigua* with MET. However, little is known about the interaction relationship between *EcRA* and MET.

*EcRA* was an important gene in the ecdysone signaling pathway, and knock down of *EcRA* in *S. exigua* increased the sensitivity to MET. Similarly, miR-189942 suppressed *PxEcR-B* expression and influenced the tolerance of *P. xylostella* to fufenozide [33]. In this study, we used an injection RNAi technique to determine that the expression of *EcRA* significantly decreased after ds*EcRA* injection compared with the control. Moreover, we applied gene silencing followed bioassay to evaluate the role of *EcRA* in the MET toxicity to *S. exigua*. After the *EcRA* was knocked down, the results of the bioassay showed that mortality of *S. exigua* was increased when exposed to MET. It indicates that *EcRA* involves in the detoxification of MET. Overall, these findings indicate that *EcRA* plays an important role in regulating the toxicity of MET, but that crosstalk between the MET and ds*EcRA* treatments for the genes in the ecdysone signaling pathway requires further study.

In this study, knockdown of *EcRA* influenced the expression of genes in the ecdysone signaling pathway. Similarly, the mRNA level of 20E signaling genes was significantly changed after the knockdown of *EcR* [34,35,36,37,38]. However, the expression of *E74* differed from previous research. There are two possible reasons for this. First, *Leptinotarsa decemlineata* and *Henosepilachna vigintioctopunctata* belong to the order Coleoptera, while *S. exigua* belongs to the order Lepidoptera, and the structure and composition could differ among the three pests. Second, MET affects the expression of *E74*. After the injection of ds*EcRcom*, the chitin contents in the cuticle significantly decreased [14]. In our research, the expressions of *G6PI*, *UAP*, and *CHSA* significantly changed in *S. exigua* treated with *MET-dsEcRA*. The expression levels of *SeTre-1, SeG6PI*, *SeUAP*, and *SeCHSA* were significantly reduced when ds*EcRcom* was injected at the larval stage of *S. exigua* [14]. 

Overall, our study reveals the gene expression of the ecdysone signaling pathway in *S. exigua* treated with different doses of MET, and that knocking down *EcRA* affects mortality of MET and the ecdysone signaling pathway. These results suggest that *EcRA*, as an important gene in ecdysone signaling pathway, plays an important role of totoxic process of MET to the target pest. Therefore, our findings offer a deep insight into interaction of *EcRA* and MET, and the action mechanisms of MET.

## 4. Materials and Methods

### 4.1. Chemicals

The insecticide used in this study was Methoxyfenozide (MET) (97% purity), which was provided by Anyang Quanfeng Biotechnology Co., Ltd. (Quanfeng Biotechnology Co., Ltd, Anyang, China). All compounds were technical grade materials, and stock solutions of 100 mg/L of technical grade materials were prepared using acetone as the solvent [23].

### 4.2. Insect Rearing

*S. exigua* larvae were collected from the cotton fields of Anyang, Henan, China, and had been raised for at least 30 generations in a greenhouse before the bioassay [39]. The larvae were maintained at (28 ± 1) °C with a photoperiod of 16:8 (L:D) H and 70% relative humidity. Adults were fed with a 10% (*w*/*v*) honey solution.

### 4.3. Sample Collection and RNA Isolation

The MET was dissolved in acetone and diluted with DEPC water to the desired concentration. L3 larvae were starved 4 h and placed into 24 orifice tissue culture plates, containing the artificial diet, via the drug–membrane method. A total of 100 μL different sublethal doses of MET (LC_10_ = 7.00 ng/cm^2^, LC_30_ = 21.00 ng/cm^2^, LC_50_ = 38.20 ng/cm^2^) was added to every orifice, and 100 μL of DEPC water was used as a control group [23]. The feed conditions of *S. exigua* were not changed.

After 72 h of MET induction, samples of the L4 larvae were taken from all treatments. Larvae were fixed and dissected, after which the cuticles were collected with tweezers. The cuticles were cleaned and placed in a 0.7% saline solution. The filter paper absorbed the residual saline from the cuticle. Biological samples were performed in triplicate, with each comprising one L4 larva cuticle that was released in a 1.5 mL tube. Total RNA (LC_10_, LC_30_, LC_50_, and DEPC water) was isolated from the cuticle tissues using TRIzol Reagent (Invitrogen, Carlsbad, CA, USA) according to the manufacturer’s instructions. Total RNA was stored at −80 °C.

### 4.4. Transcriptome Data Analysis

Approximately 1 μg of total RNA (LC_30_ and DEPC water), obtained as described above, were isolated using Poly (A) mRNA with poly-T oligo attached magnetic beads (Invitrogen). Total RNA was sent to MAJORBIO CLOUD for cDNA library construction and transcriptome sequencing. The library was constructed using an Illumina TruseqTM RNA sample prep kit and the software was used to assemble all of the clean data from scratch. The reads were decomposed, after which we built the k-mer (default k = 25), selected the seed k-mer, and extended both sides to form a contig. Overlapping contigs were clustered to form components, and each component became a set of possible representations of mutable shear isoforms or homologous genes. Each component had a corresponding DE Bruijn Graph. The DE Bruijn Graph of each component was simplified to output the full-length transcript of the variable-shear subtype, and the transcript corresponding to the homologous gene was combed to obtain the splicing result file. All UniGenes and transcripts obtained by transcriptome assembly were compared with six databases (NR, Swiss-prot, Pfam, COG, GO, and KEGG), with results providing statistics on the annotation and KEGG of each database.

The RSEM program is a tool used to estimate differentially expressed genes. The FPKM (Fragments per Kilobases per Million) method reduces the influence of differences in gene length and sequencing volume on the calculated gene expression, which can be directly used to compare differences in gene expression between different samples. Raw counts were statistically analyzed using the DESeq2 software to obtain the UniGenes and Transcripts, and compare differences in expression between the groups based on certain screening conditions. 

To identify the ecdysone signaling pathway genes in *S. exigua*, we compiled all the known genes involved in this pathway based on reports on *S. exigua* and other insects and sequenced them based on their functional annotation in the transcriptome database [19,40,41]. Primers were designed according to homologous species sequences. The high amplification efficiency and melting curve are effective at confirming primers (Table S1). 

### 4.5. Sublethal Doses of MET Induction

To investigate sublethal doses of MET induction, approximately 500 ng of total RNA (LC_10_, LC_30_, LC_50_, and DEPC water), prepared according to the above section, were selected to synthesize cDNA. The cDNA was synthesized from total RNA using Evo M-MLV Reverse Transcriptase Kit (AG, Changsha, China). The cDNA synthesized mixture (10 μL) contained 500 ng total RNA, 1 μL gDNA Clean Reagent, 2 μL 5× gDNA Clean Buffer, and RNase Free water to 10 μL. The reaction conditions were 42 °C for 2 min, and 4 °C for 10 min. The mix of step one contained 1 μL Evo M-MLV RTase Enzyme Mix, 5 μL 5× RTase Reaction Buffer Mix I, 1 μL RT Primer MIX, and 4 μL RNase Free water to 20 μL. The cDNA was synthesized following these cycling conditions: 15 min at 37 °C, 5 s at 85 °C, and 10 min at 4 °C. All cDNA was stored at −20 °C. 

### 4.6. Double-Stranded RNA (dsRNA) Synthesis

The interference primers were subjected to biosynthesis according to the T7 RNA polymerase promoter (Table S1). The DNA templates for ds*RNA* synthesis were PCR-amplified using primer sets containing the T7 promoter sequence from previously mentioned cDNA. The interference primers were synthesized according to the T7 RNA polymerase promoter. Sequences of ds*RNA* were compared with sequences of the ecdysone signaling pathway genes in the transcriptome data. A 565 bp fragment of the target gene (EcRA, ADK66917.1) and a 467 bp fragment of the green fluorescent protein gene (GFP, ACY56286.1) were amplified using specific primers [42]. PCR products were analyzed on 1% agarose gel, after which the PCR products were cloned and sequenced to confirm their identities. The resulting PCR products were then purified with a DNA cleanup kit and used as templates for in vitro transcription using the Megascript T7 transcription kit (Invitrogen, Carlsbad, CA, USA) according to the manufacturer’s instructions. ds*RNA* was amplified using primers with the T7 RiboMAX^TM^ Express RNAi system. The resulting ds*RNA* was dissolved in DEPC water and quantified at 260 nm using a spectrophotometer Nanodrop2000C (Thermo Fisher Scientific, Carlsbad, CA, USA). The diluted ds*RNA* was used immediately or stored in 10 μL aliquot at −80 °C.

### 4.7. RNA Interference (RNAi) and Bioassays

The one μg ds*RNA* was injected into L3 larva (molt < 3 h) using a microsyringe (Hamilton, Reno, NV, USA). After the injection, the L3 larvae were placed into 24 orifice tissue culture plates containing the artificial diet via the drug–membrane method; 100 μL of MET (LC_30_ = 21.00 ng/cm^2^) was added to each orifice. The control was injected with ds*GFP*. All treatments contained MET-ds*GFP* and MET-ds*EcRA*. Every treatment was replicated five times with 120 larvae, for a total of 240 treated larvae. After the successful knockdown of *EcRA*, the mortality of *S. exigua* larvae to MET was calculated after 72 h.

We collected the surviving L3 larvae following treatment at 12 h, 24 h, and 48 h, and the L4 larvae following treatment at 72 h. The larval cuticles were dissected, and total RNA extracted. The cDNA was synthesized from total RNA and stored at −20 °C.

### 4.8. Expression of Ecdysone Signaling Pathway Genes Regulated by MET-dsEcRA

In order to analyze the expression of ecdysone signaling pathway genes regulated by MET-ds*EcRA*, survival L4 larvae treated with MET-ds*GFP* or MET-ds*EcRA,* were collected in Section 4.7. Total RNA was extracted, cDNA was synthesized and stored at −20 °C.

### 4.9. qPCR Assays

The expression of *EcRA*, *USP*, *E74*, *HR3*, *HR4*, *FTZ-F1*, *HR38*, *TPS*, *TREA*, *HK*, *G6PI*, *GFAT*, *PGM*, *UAP*, and *CHSA* were analyzed after the larvae were treated with sublethal doses of MET and MET-ds*RNA* via qPCR. The interference efficiency of ds*EcRA* was performed by qPCR from 12 h to 72 h. The qPCR program was set to the following cycling conditions: 30 s at 95 °C, 40 cycles of 5 s at 95 °C, 10 s at 60 °C, and 25 s at 72 °C. The reference genes were the *elongation factor* (*SeEF*) and *Glyceraldehyde-3-phosphate dehydrogenase* (*SeGAPDH*) [43]. All samples were performed in triplicate. Target genes were calculated using the relative quantitative method (2^−^^ΔΔCT^). 

### 4.10. Statistical Data Analysis

All the results in the experiment were presented as the mean ± standard error (SE). Significant differences were determined by Student’s *t*-test, or one-way ANOVA, followed by a least significant difference test for mean comparison. The bioassay data were analyzed using IBM SPSS Statistics 20 (SPSS, Chicago, IL, USA). The primers were designed in Beacon Designer 7.0 software (Premier Biosoft, Palo Alto, CA, USA) and the pictures were composite using GraphPad Prism 7.0 (GraphPad Software Inc., San Diego, CA, USA).

## Figures and Tables

**Table 1:** Date control statistics of transcriptome of *S. exigua.*

Sample	Raw Reads	Raw Bases	Clean Reads	Clean Bases	Error Rate (%)	Q20 (%)	Q30 (%)	GC Content (%)
Control group 1	44,923,180	6,783,400,180	44,335,152	6,636,272,205	0.0236	98.66	95.48	46.75
Control group 2	46,298,736	6,991,109,136	45,854,460	6,864,878,635	0.0230	98.88	96.17	49.38
Control group 3	46,272,298	6,987,116,998	45,733,494	6,851,482,844	0.0231	98.85	96.06	49.08
MET (LC_30_) 1	48,311,340	7,295,012,340	47,908,742	7,186,907,127	0.0227	99.01	96.49	49.14
MET (LC_30_) 2	49,407,600	7,460,547,600	48,903,418	7,321,874,857	0.0229	98.93	96.28	49.00
MET (LC_30_) 3	48,680,700	7,350,785,700	48,177,980	7,222,758,714	0.0229	98.91	96.21	48.38

**Table 2:** Ecdysone signaling pathway of DEGs.

UniGenes	Gene Bank	Homologous Genes	Log_2_FC	Padjust	Organisms
TRINITY_DN68411_c0_g1	ACD39740.1	*ultraspiracle protein* (*USP*)	1.43053001	0.000710809	*S. exigua*
TRINITY_DN68123_c0_g1	XP_022816977.1	probable nuclear *hormone receptor HR3* isoform X6 (*HR3*)	1.319971145	0.407707359	*S. litura*
TRINITY_DN67384_c0_g4	GU296540.1	*ecdysone receptor A* (*EcRA*)	0.511687018	0.509814187	*S. exigua*
TRINITY_DN66888_c3_g2	XP_022822951.1	nuclear *hormone receptor FTZ-F1* beta (*FTZ-F1*)	−0.85620132	0.101532649	*S. litura*
TRINITY_DN35412_c0_g1	XM_022967707.1	PREDICTED: *ecdysone-induced protein 74EF* (*E74*)	−0.86981036	1	*S. litura*
TRINITY_DN62913_c0_g1	XP_021187825.1	*hormone receptor 4* isoform X1 (*HR4*)	−3.19753521	8.62 × 10^−9^	*H. armigera*
TRINITY_DN68583_c0_g4	XP_022820857.1	probable nuclear *hormone receptor HR38* (*HR38*)	−6.28201224	3.90 × 10^−40^	*S. litura*
TRINITY_DN65723_c0_g2	ACV97159.1	*glucose-6-phosphate isomerase* (*G6PI*)	1.468813444	0.000855695	*S. exigua*
TRINITY_DN66137_c0_g2	ABY86218.1	*trehalase-1* (*TREA*)	0.83888701	0.486117572	*S. exigua*
TRINITY_DN67124_c0_g6	ABM66814.2	*trehalose 6-phosphate synthase* (*TPS*)	−0.15968318	0.840417477	*S. exigua*
TRINITY_DN63532_c0_g3	XP_022834981.1	*hexokinase type 2 isoform X6* (*HK*)	−0.54980536	0.337950088	*S. litura*
TRINITY_DN68071_c2_g3	XP_022827210.1	*Phosphoacetyl glucosamine mutase* (*PGM*)	−0.81471571	0.132596543	*S. litura*
TRINITY_DN65476_c0_g2	AAZ03545.1	*chitin synthase A* (*CHSA*)	−1.99147489	2.42017 × 10^−8^	*S. exigua*
TRINITY_DN66504_c1_g4	XP_022821069.1	*glutamine--fructose-6-phosphate aminotransferase* (*GFAT*)	−4.07457569	3.01573 × 10^−29^	*S. litura*
TRINITY_DN67977_c1_g2	ACN29686.1	*UDP-N-acetylglucosamine pyrophosphorylase* (*UAP*)	−5.02829161	1.86507 × 10^−40^	*S. exigua*

## Data Availability

The raw RNA-seq data are available in the National Center for Biotechnology Information (NCBI) under SRA accession number: PRJNA705838.

## Supplementary Materials

The following are available online at https://www.mdpi.com/article/10.3390/ijms22169080/s1.
