# Supplementary material for: Combined Transcriptomic Analysis and RNA Interference Reveal the Effects of Methoxyfenozide on Ecdysone Signaling Pathway of Spodoptera exigua
Source: Int J Mol Sci. 2021 Aug 23;22(16):9080. doi: 10.3390/ijms22169080 (PMC8396458; doi:10.3390/ijms22169080)
Supplement: Supplementary file 1 [file ijms-22-09080-s001.zip › ijms-1319313-supplementary.pdf]

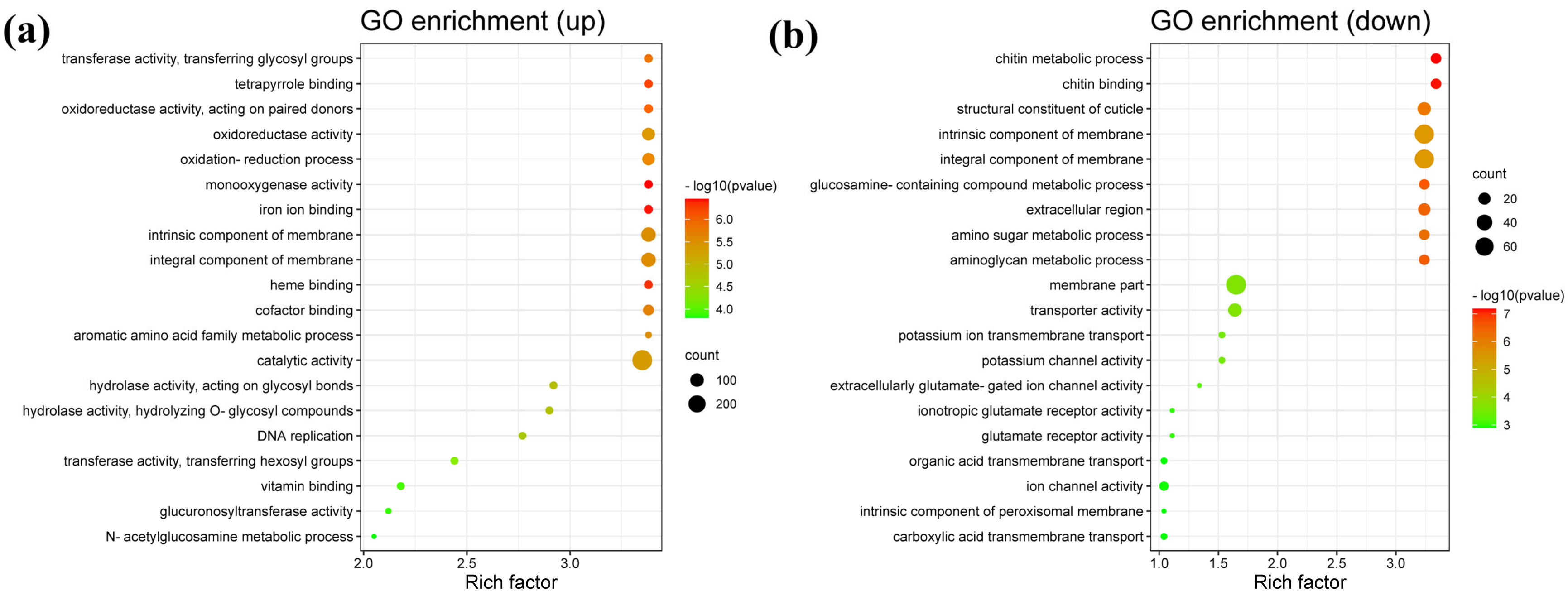

Figure S1. Comparison of up- and down-regulated Gene ontology(GO) enrichment terms of differentially expressed genes (DEGs) of *S. exigua* after exposure of MET.

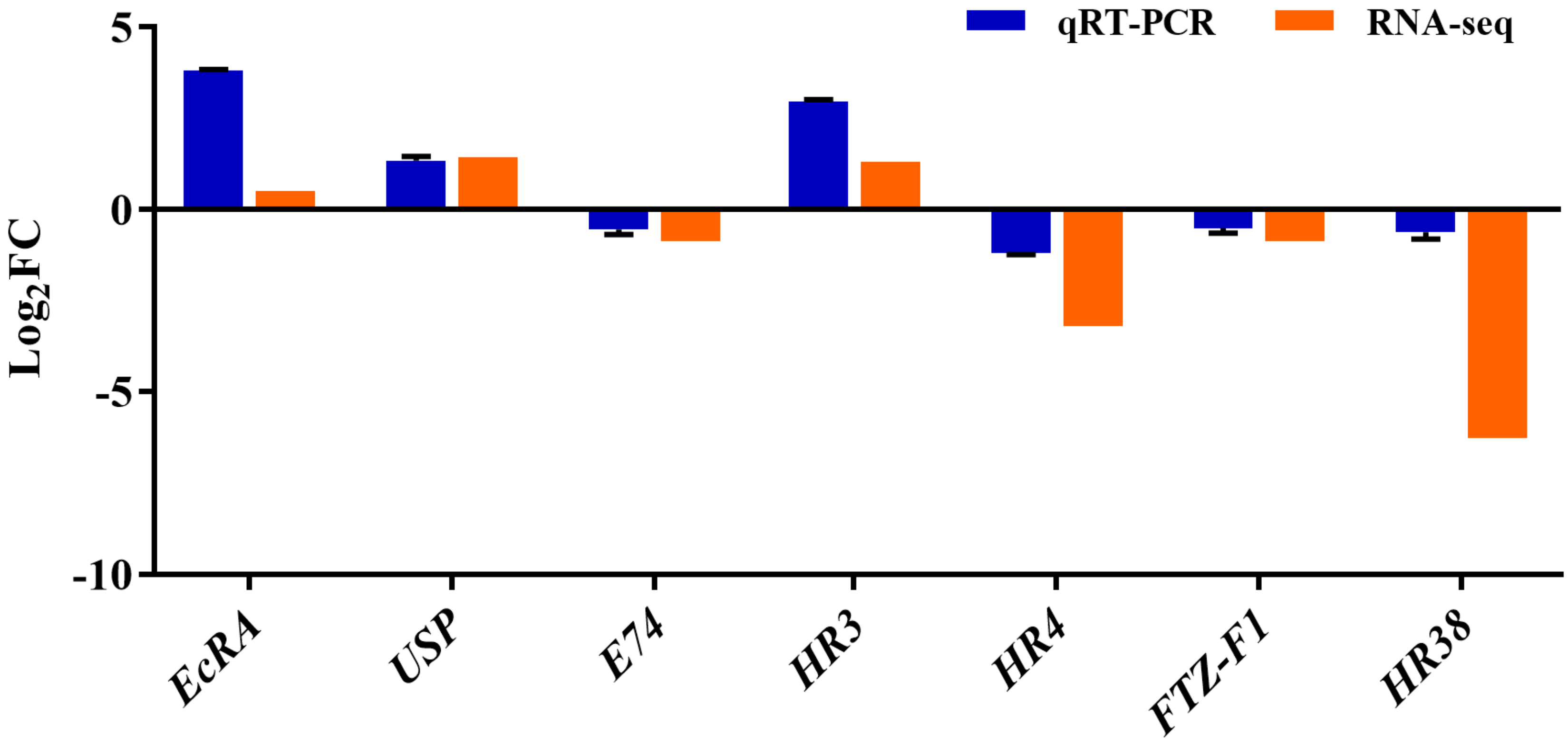

Figure S2. Prove transcriptome data according qPCR (Blue represent transcriptome date, orange represent qPCR). The bars represent the average (±SE) of biological repeats. Expression ratios of 7 candidate genes in log_2FC (The fold changes of the genes were calculated as the value of each FC comparison and are shown on the y-axis. Blue bars represent gene expression data ($2^{-\Delta\Delta Ct}$) that was obtained by qPCR analysis).

Table S1 Primer designer

Primer	Equation	R^2	Amplification efficiency	Forward Primer(5'-3')	Reverse Primer(5'-3')
USP	y=-3.3143x+34.567	0.99	97%	GTAGGTCATTGAACTGTAG	GACACCTACCTGACGATTA
HR3	y=-3.3269x+32.564	0.99	95%	GGTGATATAAGCAAGGTC	GTAGGTCATTGAACTGTAG
EcRA	y=-3.3593x+33.527	0.99	98%	GGAGCTATGTCTCGTCTG	AATCCTTTACACCCTTCACA
FTZ-F1	y=-3.2212x+37.386	0.99	104%	AACACAACGGAAGCGCTGCC	GGTGACGACTCTGGCGATGA
HR4	y=-3.3597x+36.638	0.98	98%	AGATACCAGCACGTCGTCGC	GATGGCAAGCAGGCGATGGT
HR38	y=-3.3765x+32.309	0.98	98%	AATGGACGAAGACTGCTA	ATGGAAGGTTGATGATATTGAT
E74	y=-3.3888x+31.016	0.99	97%	CACTGTTTCCTGGTTGTTGC	AGTCAGCAACAGCAACAACA
TPS	y=-3.4958x+32.035	0.99	93%	CGCTACACTTGAGTTGAA	ATTGGATGCTATTGAGGTT
TREA	y=-3.4174x+30.393	0.98	96%	ATTGGTGTATTGGTAGTAAGT	GAAGCAGGTGGTATTATCA
HK	y=-3.3729x+34.474	0.99	98%	TTCTCTTGATTCTTCCAT	CACACGCTCTTATTATAG
G6PI	y=-3.3048x+34.424	0.98	101%	TACCTGACTTCTCTAACT	ATCTTACTAGCCAACTTC
GFAT	y=-3.3493x+30.497	0.99	99%	TCCATTGACATTCCATTA	ACTTATTGACATACGGTAA
PGM	y=-3.2192x+31.904	0.99	104%	GGACTGAAGATATTGTTAGAG	GAATACTGCTTGACATACTT
UAP	y=-3.3597x+34.668	0.98	98%	GGTGGTATTGTAGAAGAC	GGACTAATTGTGAAGACT
CHSA	y=-3.3915x+33.309	0.98	97%	CGCTCATACAGTTCTATA	CTTCATTCAACATCCATT
SeGAPDH	-	-	-	GACAACCACTCATCTATCTTCG	AACATTTATCTCTACAACGCAATC
SeEF	-	-	-	TGATGCCACAGACAAGAG	CCATTTCCACAAGTTCTACC
ds*EcRA*	-	-	-	ggatcctaatacgactcactatagg	ggatcctaatacgactcactatagg
	-	-	-	GTGTCGGTTGAAGAAATGTCT	CGCAACATCATCACCTCACT
dsGFP	-	-	-	ggatcctaatacgactcactatagg	ggatcctaatacgactcactatagg
	-	-	-	AAGGGCGAGGAGCTGTTCACCG	CAGCAGGACCATGTGATCGCGC
